# Vagus Nerve Stimulation Attenuates Cerebral Ischemia and Reperfusion Injury via Endogenous Cholinergic Pathway in Rat

**DOI:** 10.1371/journal.pone.0102342

**Published:** 2014-07-18

**Authors:** Ying Jiang, Longling Li, Bin Liu, Yanhong Zhang, Qian Chen, Changqing Li

**Affiliations:** Department of Neurology, The Second Affiliated Hospital of Chongqing Medical University, Chongqing, China; University of California, Los Angeles, United States of America

## Abstract

Inflammation and apoptosis play critical roles in the acute progression of ischemic injury pathology. Emerging evidence indicates that vagus nerve stimulation (VNS) following focal cerebral ischemia and reperfusion (I/R) may be neuroprotective by limiting infarct size. However, the underlying molecular mechanisms remain unclear. In this study, we investigated whether the protective effects of VNS in acute cerebral I/R injury were associated with anti-inflammatory and anti-apoptotic processes. Male Sprague-Dawley (SD) rats underwent VNS at 30 min after focal cerebral I/R surgery. Twenty-four h after reperfusion, neurological deficit scores, infarct volume, and neuronal apoptosis were evaluated. In addition, the levels of pro-inflammatory cytokines were detected using enzyme-linked immune sorbent assay (ELISA), and immunofluorescence staining for the endogenous “cholinergic anti-inflammatory pathway” was also performed. The protein expression of a7 nicotinic acetylcholine receptor (a7nAchR), phosphorylated Akt (p-Akt), and cleaved caspase 3 in ischemic penumbra were determined with Western blot analysis. I/R rats treated with VNS (I/R+VNS) had significantly better neurological deficit scores, reduced cerebral infarct volume, and decreased number of TdT mediated dUTP nick end labeling (TUNEL) positive cells. Furthermore, in the ischemic penumbra of the I/R+VNS group, the levels of pro-inflammatory cytokines and cleaved caspase 3 protein were significantly decreased, and the levels of a7nAchR and phosphorylated Akt were significantly increased relative to the I/R alone group. These results indicate that VNS is neuroprotective in acute cerebral I/R injury by suppressing inflammation and apoptosis via activation of cholinergic and a7nAchR/Akt pathways.

## Introduction

Acute cerebral ischemia triggered by interruption of blood flow can cause rapid activation of a variety of detrimental cellular processes, including excitotoxicity, oxidative and nitrosative stress, cortical spreading depolarization, inflammation, necrosis, and apoptosis [1]–[2]. It is known that revascularization of occluded blood vessels is the most effective treatment for patients with acute ischemic stroke [3]. However, reperfusion may cause secondary injury via mitochondrial dysfunction, excessive release of glutamate, and overproduction of pro-inflammatory mediators and reactive oxygen species (ROS). These factors directly influence the prognosis of stroke patients [4], and controlling excessive inflammatory response is an important therapeutic challenge.

Since its approval for clinical use by the Food and Drug Administration (FDA) in 1997, vagus nerve stimulation (VNS) has been a safe and effective treatment for refractory partial epilepsy seizures [5]–[6] and resistant depression [7]. Its therapeutic potential in other applications, including pain [8], obesity [9], cognitive impairment [10], and anxiety [11] have been explored as well. Outside the CNS, VNS has been reported to have an anti-inflammatory effect [12]. In rat cardiomyocytes under hypoxia conditions, VNS activates the anti-apoptotic process involving increased phosphorylation of Akt via upregulation of acetylcholine (Ach) release [13]. Emerging evidence demonstrated that a brief VNS initiated 30 min after both transient and permanent cerebral ischemia significantly improved neurological function deficits and reduced infarct volume in I/R rats [14]. However,the molecular mechanisms underlying VNS-mediated neuroprotection are still unknown.

In this study, we evaluated the neuroprotective effects of VNS in an animal model of acute cerebral I/R. We assessed neurological deficit scores, infarct volume, and neuronal apoptosis. In addition, we measured the level of pro-inflammation cytokines, activation of the cholinergic anti-inflammatory pathway, and the a7nAchR/Akt signaling pathway involved in preventing neuron death under acute cerebral I/R conditions.

## Materials and Methods

The experimental protocols were performed in strict accordance with the Guidelines for the Care and Use of Laboratory Animals approved by the Institutional Ethics Committee of Chongqing Medical University (Permit No. SCXK (Chongqing) 2007-0001) and the State Science and Technology Commission of China. Animal experiments were performed at the Laboratory Animal Management Committee of Chongqing Medical University. Male Sprague-Dawley (SD) rats (250–350 g, n = 158) were obtained from Experimental Animals Center of Chongqing Medical University and housed at 20–25°C and 60% humidity with a 12 h light/dark cycle, and free access to food and water. Animals were randomly assigned to one of three groups (n = 8 rats/group): sham+VNS group; I/R group; and I/R+VNS group. There was no significant difference in weight among the groups.

### The focal cerebral ischemia and reperfusion model

The focal cerebral ischemia model was established according to the intraluminal occlusion technique as previously described by Koizumi et al [15]. First, animals were anesthetized with 10% chloral hydrate (350 mg/Kg). After a midline neck incision with the animal in supine position, the right common carotid artery (CCA) and internal carotid artery (ICA) were sequentially exposed from adjacent nerves and tissue. With the right external carotid artery (ECA) ligated, microaneurysm clips were placed at both ICA and ECA, a small incision was made on the ECA stump near the carotid bifurcation, and a nylon filament coated with silicone (diameter = 0.31–0.32 mm) was inserted into the right internal carotid artery about 9–10 mm. The animals were then placed into a stereotaxic frame, and a laser doppler flowmeter probe (Peri Flux system 5000; Perimed, Stockholm, Sweden) monitored changes in cerebral blood flow (CBF) in the right middle cerebral artery region in order to confirm successful middle cerebral artery occlusion (MCAO). When the nylon filament was inserted approximately 18–22 mm, a silk suture was then tightened around the ECA stump and nylon filament. Reperfusion was achieved after removal of the nylon filament 2 h after occlusion. The standard I/R model was defined as a decrease in cortical CBF to 70–80% of baseline during the first 30 min and >70% flow recovery within the initial 10 min of reperfusion. Animals that did not meet these requirements or those without neurological deficits were excluded from the study. Throughout the duration of the experiment, animals were anesthetized and body temperature was maintained at 37±0.5°C with a heating pad. The caudal ventral artery of the tail (coccygeus medial artery, CVA) was exposed, and 24 G catheters (Becton Dickinson and Company, Franklin Lakes, NJ, USA) were inserted and connected to a Datex AS3 physiological monitoring instrument (Communications Specialties, Inc., Ronkonkoma, NY, USA) through heparin-filled pressure transducers and sampled for blood gas analysis. Invasive blood pressure (BP) monitoring occurred at five time points: baseline (BS), before VNS (BVNS) (0–30 min after MCA occlusion), during VNS (DVNS) (30–90 min after MCAO), after VNS and before reperfusion (AVNS), and after reperfusion (AR); and heart rate (HR) was measured at the same time points during the duration of the experiment. Blood gases were measured at three points: BVNS, DVNS, and AR (ABL700, Radiometer, Bronshoi, Denmark).

### Electrical stimulation of right cervical VN

**Figure 1:**
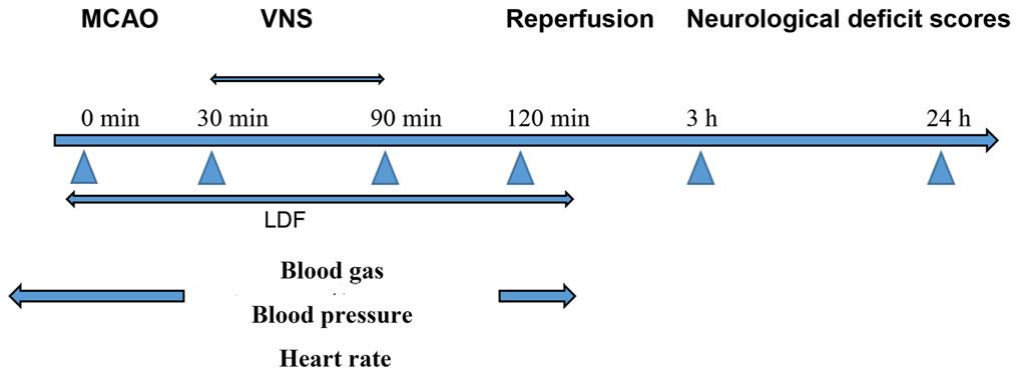
The Experimental Protocol. I/R and I/R+VNS groups underwent transient MCAO surgery. In the I/R+VNS and sham+VNS group, VNS was initiated at 30 min after occlusion and repeated every 5 min for 1 h. At 120 min, I/R and I/R+VNS groups underwent reperfusion. During the experiment, the mean CVA, HR, and blood gas were measured at the designated time points. Laser doppler flowmetry monitored the changes of right cerebral middle artery blood flow, and temperature was maintained at approximately 37°C. Neurological deficit scores assessment was performed at 3 and 24 h after reperfusion.

Animals received VNS 30 min after MCAO with a Grass Model S48 stimulator (Grass Technologies, Warwick, RI, USA). VNS consisted of 30 s, 20 Hz trains of 0.5 mA, 0.5 ms pulses delivered every 5 min for a total of 60 min [16]. The stimulating electrodes were self-constructed, as previously described [17], and were composed of two polyethylene coated curved silver wires held 1.5 mm apart by a solid bar. Following a ventral midline incision made on the neck, muscles were retracted and the right carotid sheath was exposed. The right cervical VN was carefully dissected from the carotid sheath, and the exposed wires inside of the curve of the stimulating electrode were placed on the nerve. Electrodes were then sutured to the sternocleidomastoid muscle and wrapped around the right cervical nerve. Identical procedures were performed in the I/R+VNS and sham+VNS group with the exception of the occlusion in the sham+VNS group (Fig.1). Two rats died during the stimulation process because of cardiac arrest (I/R+VNS group), and one (I/R group) died due to the suffocation.

### Evaluation of neurological function scores

Neurological function of the rats (n = 8 rats/group) was evaluated 3 and 24 h after surgery according to the criteria of a 5-point rating scale: 0, no deficit; 1, failure to extend the left forepaw; 2, decreased grip strength of left forepaw; 3. circling to left after pulling the tail; 4, spontaneous circling [18].

### Cerebral infarct volume measurement

At 24 h after assessment of neurological deficit scores, rats (n = 8 for each group) were deeply anesthetized and sacrificed, and fresh brains were rapidly removed and frozen at −20°C for 20 min. The brains were coronally sectioned into 2- mm thick sections with a stainless steel mold, and sections were stained in a 2% 2,3,5-triphenyltetrazolium chloride (TTC, Sigma-Aldrich, St. Louis, MO, USA) solution for 30 min at 37°C in the dark, and fixed in 4% paraformaldehyde (PFA) in 0.1 M PB. Stained slices were photographed with a digital camera and analyzed with Image J software (NIH Image, Bethesda, MD, USA).The infarct volume was calculated as described previously [19]. The percentage of infarct volume was calculated as the (normal hemisphere volume minus non-infarct volume of infarct side) over the normal hemisphere volume times 100%.

### TUNEL staining

DNA fragmentation, a marker of apoptosis, was measured using TdT mediated dUTP nick end labeling (TUNEL) staining with an In Situ Cell Death Detection Kit® (Roche, Basel, Switzerland), as previously described [20]. At 24 h after reperfusion, rats (n = 10 for each group) were first anesthetized with 10% chloral hydrate (350 mg/Kg) and serially transcardially perfused with 150 ml phosphate-buffered saline (PBS) and 150 ml 4% paraformaldehyde until all limbs became stiff. The brains were removed, post-fixed in 4% paraformaldehyde for 24 h, and then embedded in paraffin for slicing 5 µm sections. Briefly, the sections were post-fixed in ethanol-acetic acid (2∶1), rinsed, incubated with 1% proteinase K (in 50 mM Tris/5 mM EDTA buffer) for 15 min, rinsed, incubated in 3% H_2_O_2_, permeabilized with 0.5% Triton X-100, and rinsed again. Subsequently, sections were incubated in the TUNEL mixture for 1 h at 37°C, rinsed, and visualized using a converter-POD with 0.03% 3,3′-diaminobenzidine, counterstained with hematoxylin/eosin (HE). Five different random high-power fields (400X magnification) in peri-infarct region were counted for each condition using a light microscope, and TUNEL-positive apoptotic cells appeared as brown nuclear or cytoplasmic staining. The numbers of TUNEL positive cells from three slices per brain were averaged. Cell counting was performed by a pathologist blinded to the experiment condition.

### Enzyme-linked immune sorbent assay

Rats (n = 15 for each group) were sacrificed 24 h after neurological functional assessment, and the right ischemic penumbra cortices (n = 5 for each group/time) were isolated and rinsed with ice-cold physiological saline for enzyme-linked immune sorbent assay (ELISA) and immunoblotting. Using ELISA, the concentrations of TNF-a, IL-1β, and IL-6 in ischemic cortex tissue homogenates were measured according to the manufacturer's instructions (R&D systems, Minneapolis, MN, USA). The experiments were performed in triplicate.

### Double immunofluorescence staining for microglia and a7nAchR

Rats (n = 8 for each group) were anesthetized 24 h after surgery and serially transcardially perfused with 150 ml PBS and 150 ml 4% paraformaldehyde in 0.1 mol/L PBS (pH 7.4). Brains were post-fixed in the same fixative overnight and infiltrated with 30% sucrose at 4°C. Frozen brains were sliced into 10 µm thick coronal sections, permeabilized with 0.4% TritonX-100 for 10 min, blocked in 10% normal donkey serum for 30 min, and incubated with primary antibodies (rabbit anti-a7nAchR, 1∶500, Abcam, Cambridge, UK and goat anti-Iba-1, 1∶500, Novus Biologicals, Littleton, CO, USA) for 16 h at 4°C. The next day, sections were rewarmed at 37°C for 1 h, washed with PBS for 15 min, incubated with secondary antibodies (AMCA-conjugated AffiniPure Donkey Anti-Rabbit IgG 1∶200 and FITC-conjugated AffiniPure Donkey Anti-Goat IgG;1∶200, Sheng Gong,Shanghai,China) for 1.5 h at 37°C, and washed for 30 min with PBS. Sections were observed and images were captured using a Laser Scanning Confocal Microscope (LEICA TCS SP2,Wetzlar, Germany).

### Immunoblotting

As previously described, protein samples were generated from frozen cortex homogenized with lysis buffer [21]. Equal amounts of protein were loaded onto either 10% or 12% sodium dodecy1 sulfate-polyacrylamide gel electrophoresis gels (SDS-PAGE) and electrophoretically transferred onto nitrocellulose membranes. Membranes were blocked with Tris-buffered saline+0.1% Tween-2 (TTBS) plus 50 g/L nonfat milk for 2 h and then incubated with a rabbit anti-p-Akt antibody (1∶1000; Cell Signaling Technology, Beverley, MA, USA), a rabbit anti-a7nAchR antibody (1∶1000, Abcam) a mouse anti-caspase-3 antibody (1∶1000; Cell Signaling Technology), and a mouse anti-β-actin (1∶5000; Sigma) at 4°C overnight. The membranes were washed for 15 min in TBST and incubated with HRP-conjugated secondary antibodies for 2 h at 37°C. The bands were visualized using an enhanced chemiluminescence reagent kit (GE Healthcare, Chalfont St. Giles, UK), detected on X-ray film, and analyzed with the Image J (National Institutes of Health, Bethesda, MD, USA). Results were expressed as the ratio of target protein OD over β-actin OD.

### Statistical Analysis

All experimental data, except for neurological scores, were expressed as mean±standard error of the mean (SEM) and compared using a one-way analysis of variance (ANOVA) with a post hoc Tukey multiple-comparison test. The neurological scores were analyzed with Kruskal-Wallis test. P-value of less than 0.05 was considered statistically significant. All statistical analyses were carried out using SPSS, Version 17.0 (Chicago, IL, USA).

## Results

### Physiological measurements

**Figure 2:**
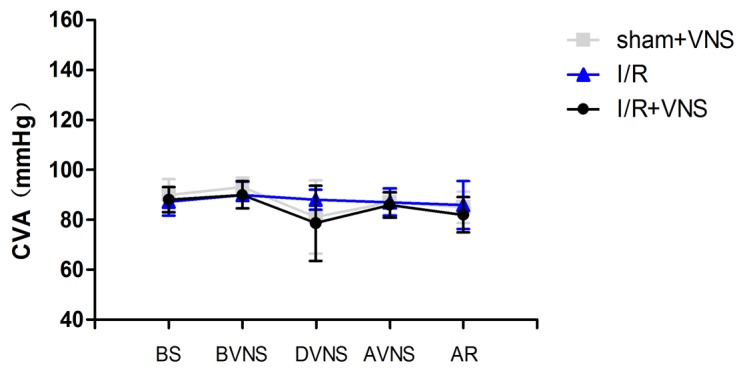
Time course of CVA during the experiment. Changes in tail arterial pressure are shown for the three groups during the experiment. The time points are as follows: baseline (BS), after occlusion but before stimulation (BVNS), during the stimulation (DVNS), after stimulation but before reperfusion (AVNS), and after reperfusion (AR). * p<0.05 relative to the time point (BVNS). #p<0.05 relative to the time point (AVNS).

As shown in Fig. 2, the mean CVA pressure in the baseline period was 90.5±6.3 mmHg in the sham+VNS group, 87.3±5.7 mmHg in the I/R group, and 88.7±5.1 mmHg in the I/R+VNS group. There were no significant changes in mean CVA pressure during the MCAO. In the sham+VNS and I/R+VNS groups, the BP decreased relative to baseline by 81.2±14.7 and 78.7±15.1 mmHg, respectively, during the 30 s stimulation period. After VNS, mean arterial pressure rapidly recovered near to baseline level.

**Figure 3:**
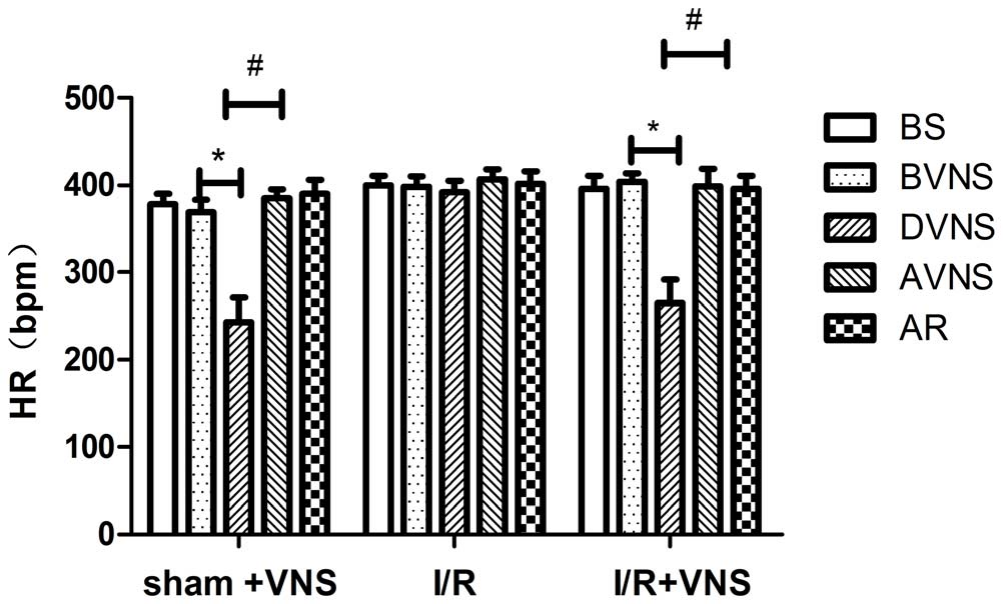
Time course of HR during the experiment. HR is plotted for the same time points as in Fig. 2. HR and BP immediately fell during each pulse train and recovered to baseline values upon cessation of the stimulation in I/R+VNS and sham+VNS groups. * p<0.05 in comparison to the time point (BVNS). ^#^p<0.05 in comparison to the time point (AVNS).

There was no significant difference in HR during baseline between the I/R+VNS group and other groups (p>0.05). HR in the I/R+VNS group decreased dramatically during the 30 s electrical stimulation, approximately 265±27 beats per minute (bpm), and HR returned immediately to normal range upon cessation of the stimulation. A similar change in HR was observed in the sham+VNS group (Fig.3). In all three groups, arterial blood gases(PH, PO_2_, and pCO_2_) remained within the normal range during the experimental period (Table.1).

**Table 1:** Blood gas analysis for all three groups.

Group	Time point	PH	PCO_2_(mmHg)	PO_2_(mmHg)
Sham+VNS	BVNS	7.40±0.02	46.3±1.0	110.5±7.1
	DVNS	7.40±0.01	45.5±1.3	119.2±10.3
	AR	7.39±0.01	47.2±2.0	115.1±8.5
I/R	BVNS	7.39±0.03	47.1±1.4	108.6±5.4
	DVNS	7.40±0.02	46.0±1.1	110.8±7.3
	AR	7.42±0.03	45.7±0.9	110.8±7.3
I/R+VNS	BVNS	7.38±0.03	45.7±0.8	115.3±6.9
	DVNS	7.40±0.03	44.2±1.2	113.4±5.2
	AR	7.38±0.01	44.9±1.1	117.9±6.2

All data are shown as mean± standard error of measurement(SEM),n = 8/group.

### Monitoring of region cerebral blood flow

**Figure 4:**
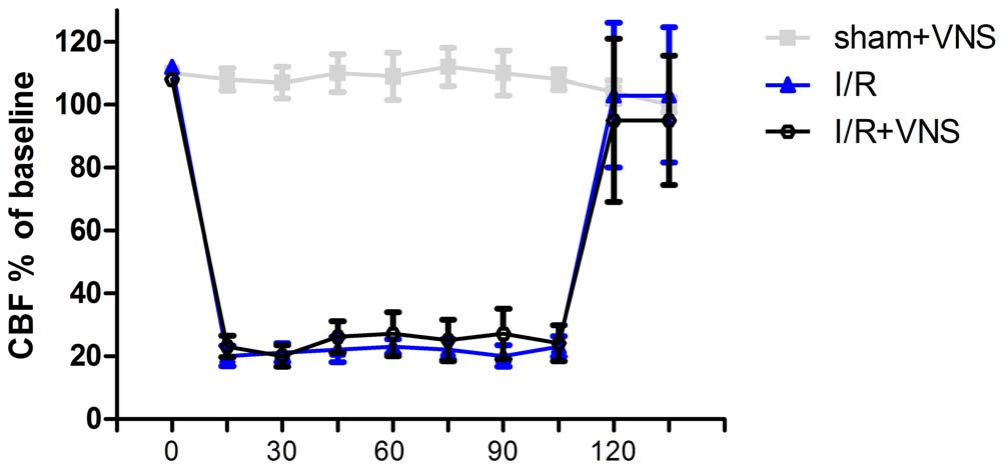
Effect of VNS on regional blood flow of right middle cerebral artery. Changes of CBF in the right middle cerebral artery territory in three group (n = 8/group). Data are shown as mean±SEM.(P>0.05).

As shown in Fig. 4, CBF of the right middle cerebral artery region was decreased by 70–80% of baseline after occlusion and returned to pre-ischemic level when the filament was removed in both I/R and I/R+VNS groups. Although there was a small increase in the I/R+VNS group relative to the I/R group during the stimulation, it was not statistical difference (p>0.05), suggesting that VNS had no significant effect on the changes of CBF in I/R rat.

### Neurological deficit scores

**Figure 5:**
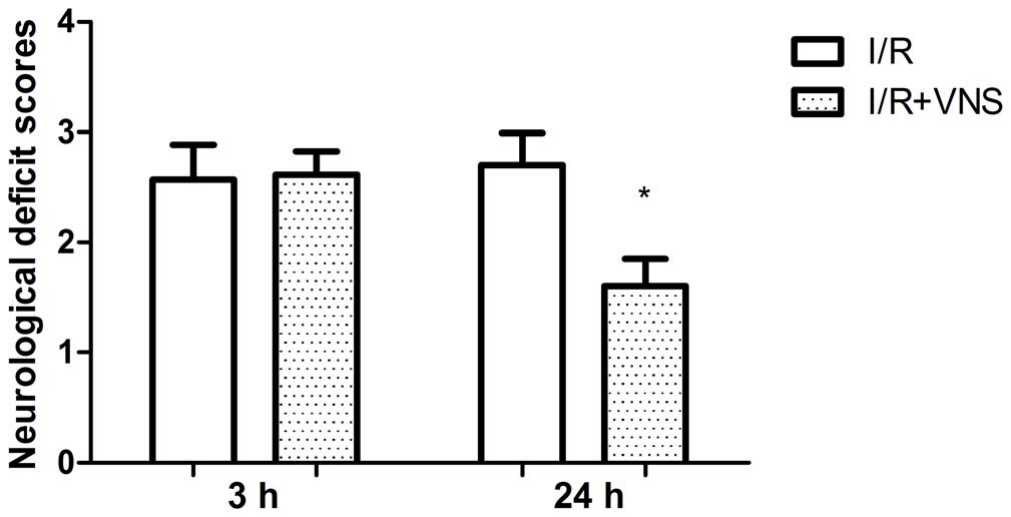
Neurological deficit scores after I/R with and without VNS. Bar graphs show the neurological deficit scores at 3 and 24 h following reperfusion in the three groups. Data are shown as mean±SEM *p<0.05.

Neurologic deficit scores were acquired at 3 h and 24 h after occlusion in the I/R and I/R+VNS groups. The scores of the I/R+VNS group were significantly lower than the I/R group at 24 h but not 3 h (Fig. 5, p<0.05), suggesting that VNS reversed neurological deficits 24 h after ischemia.

### Measurement of infarct volume

**Figure 6:**
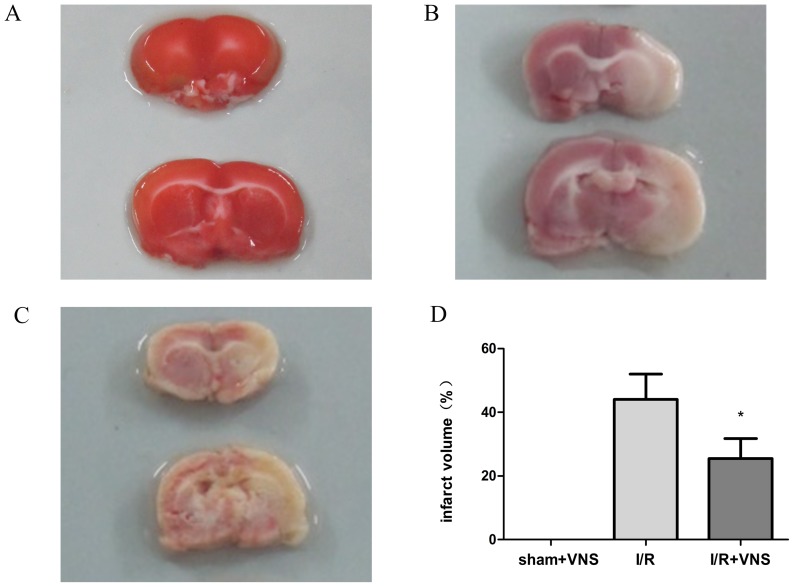
Neuroprotection induced by VNS on the infarct volume following I/R. TTC staining in the three groups at the level of bregma 0.4mm. A: sham+VNS group, B: I/R group, and C: I/R+VNS group. D. Histograms show that the relative percentage of infarct volume 24 h after acute I/R. Red area is the healthy tissue, and the white area is the infarct tissue. *p<0.05 with relative to the I/R group, #p<0.05 relative to sham+VNS group.

In order to determine the extent of brain damage 24 h following reperfusion, TTC staining was performed. As shown in Fig. 6, the infarct volume was significantly smaller in the I/R+VNS group relative to I/R group. The percentage of infarct volume of the contralateral hemisphere in the I/R+VNS treatment group was 25.4±6.3% and 44.1±8.0% for the I/R group (p<0.05). Therefore, VNS reduced the infarct volume 24 h after reperfusion.

### TUNEL staining

**Figure 7:**
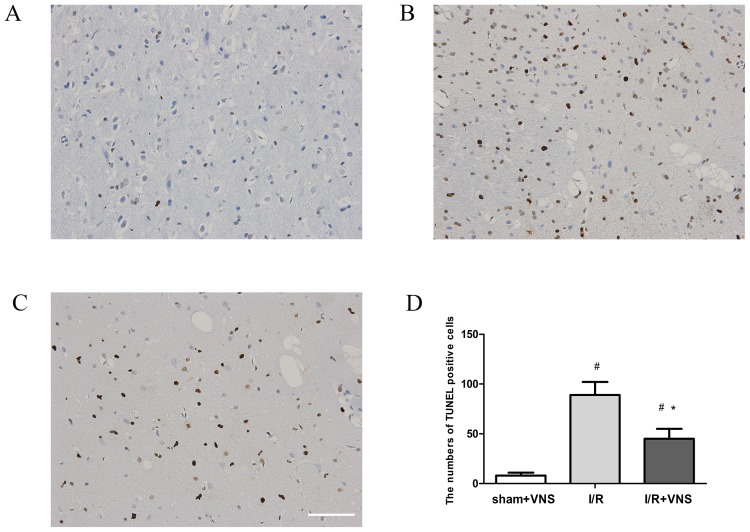
Effect of VNS on neuronal apoptosis in ischemic penumbra. Representative photomicrographs of neuron apoptosis in right ischemic penumbra cortex 24(magnification 400×). A: sham+VNS group, B: I/R group, C: I/R+VNS group. D: Quantitative analysis of the number of TUNEL-positive cells in the ischemic penumbra. Data are expressed as mean ±SEM; ^*^P<0.05 vs I/R group, #p<0.05 vs sham+VNS group, scar = 100 µm.

The number of TUNEL-positive cells in the per-infarct area of the right cortex in the I/R+VNS group was significantly less than that in the I/R group (Fig. 7). Quantitative analysis showed that VNS suppressed apoptotic responses, which may partly explain the neuroprotective effect of VNS in ischemic brain injury.

### Effect of VNS on the inflammatory cytokines

**Figure 8:**
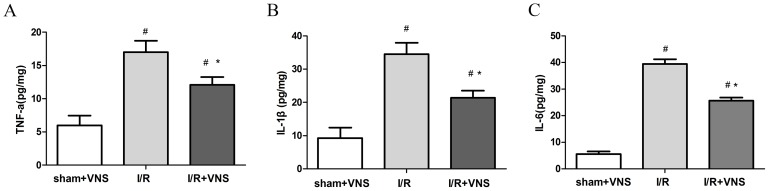
Effect of VNS on inflammatory cytokines in the ischemic penumbra at 24 h after reperfusion. The concentrations of TNF-a, IL-1β, and IL-6 of the peri-infarct region were measured by ELISA. A: TNF-a, B: IL-1β, C: IL-6 at 24 h reperfusion in the peri-infarct region. Data are presented as mean±SEM (n = 15 per group); *P<0.05 vs I/R group, #p<0.05 vs sham+VNS group.

To determine the anti-inflammatory response following transient VNS in rats with I/R injury, we measured the levels of pro-inflammatory cytokines (TNF-a, IL-1β, IL-6) in the ischemic penumbra cortex 24 h after reperfusion. As expected, the levels of TNF-a, IL-1β,IL-6 were significantly decreased in the I/R+VNS group compared with the I/R group(p<0.05, Fig. 8). This clearly showed that VNS modulated the expression of inflammatory cytokines in brain under ischemic conditions.

### Expression of a7nAchR on microglia following ischemia-reperfusion

**Figure 9:**
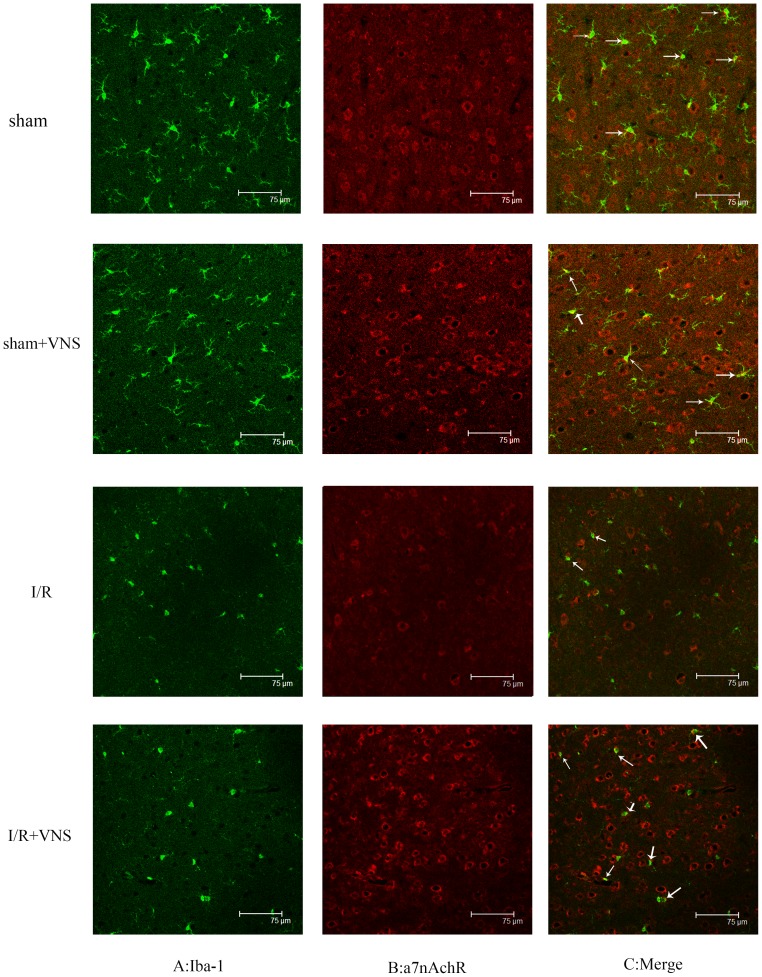
Immunostaining for a7nAchR on microglia (Iba-1) surface in ischemic penumbra. The expression of a7nAchR was reduced following I/R and increased by VNS. Underwent ischemic insults, the morphology of the microglial cell changed. A: Staining for a7nAchR in red, B: Staining (microglia) Iba-1 in green, C: merge of A and B. scare bar = 75 µm.

Under normal conditions, microglia have highly motile branches that persistently survey the CNS to detect aberrations from homeostasis. In response to brain injury, microglia reduce the complexity of their shape by shortening their branches and rapidly move to injury sites to express chemokines and growth factors. As shown in Fig. 9, microglia were activated rapidly with morphological changes consistent with brain injury in both the I/R and I/R+VNS groups. There was no significant difference between the sham and sham+VNS groups. The expression of a7nAchR on microglia in the ischemic penumbra was significantly elevated in the I/R+VNS group compared with the I/R group. These findings suggest that VNS may modulate inflammatory responses induced by I/R injury in brain via activating a7nAchR on microglia.

### Western blot analysis

**Figure 10:**
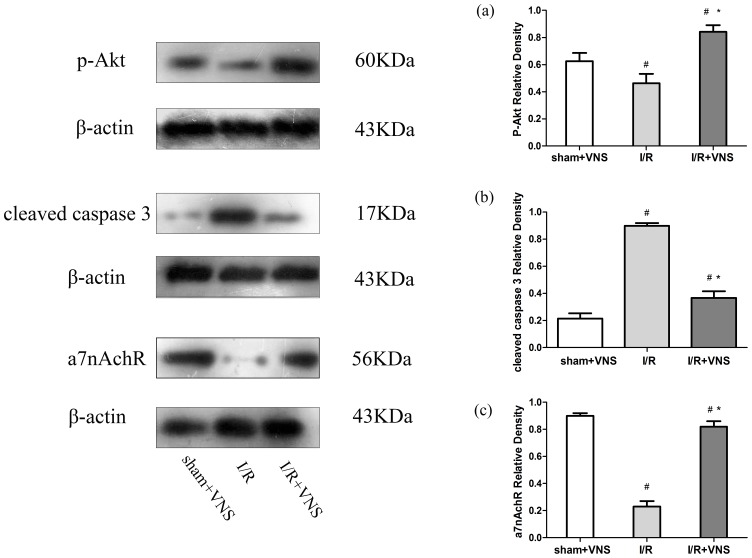
Expression of a7nAchR, P-Akt, and cleaved caspase 3 protein in the ischemic penumbra. Western blot showing the differential protein expression of a7nAchR, P-Akt, and cleaved caspase-3 24 h following reperfusion in the ischemic penumbra of rats among the three groups. β-actin is a loading control, showing equal loading of protein. A: sham+VNS group; B: I/R group; C: I/R+VNS group. (A) The density of a-7nAchR, (B) p-Akt, and (C) cleaved caspase 3 protein expression normalized to β-actin protein expression. *P<0.05 relative to the I/R group, #p<0.05 relative to the sham+VNS group, n = 8/group.

As shown in Fig. 10, protein expression of a7nAchR was significantly decreased in the I/R group relative to the sham+VNS group, and this downregulation was inhibited by VNS (P<0.05). We found that the expression of a7nAchR in brain could be regulated by VNS treatment after I/R in rats, suggesting that the neuroprotective effect induced by VNS may be partly due to the up-regulation of a7nAchR expression. To further investigate the anti-apoptotic mechanism of VNS on cerebral I/R, we also measured the protein expression of phosphorylated Akt and cleaved caspase 3 in ischemic penumbra cortex 24 h after reperfusion. Relative to the I/R group, the level of phosphorylated Akt (p-Akt) was significantly upregulated and the level of cleaved caspase-3 was noticeably decreased in I/R+VNS group (p<0.05, Fig.9). Thus, VNS may exert anti-apoptotic effect via activation a7nAchR/Akt signaling pathway in brain following cerebral I/R.

## Discussion

In this study, we found that a brief repetitive VNS could improve neurological deficit scores, attenuate cerebral infarct volume, and decrease neuronal apoptosis 24 h after reperfusion. Importantly, the neuroprotective effect is independent of any alterations in CBF [22].

Previously, it was shown that VNS could improve neurological scores and reduce infarct volume by nearly 50% 24 h after transient MCA occlusion in a rat model of I/R injury [22], suggesting a neuroprotective role of VNS in acute cerebral ischemia. Interestingly, it had been demonstrated that the neuroprotection induced by VNS could be maintained up to three weeks after the ischemic event [23]. Here, at 24 h after ischemia, neurologic deficit scores, ischemic infarct volume, and the number of TUNEL positive cells were decreased in the I/R+VNS group relative to the I/R group, consistent with another study [24]. It is our supposition, based on our and other studies, that VNS-associated neuroprotection in acute ischemic stroke is due to inhibition of inflammatory and apoptotic processes.

Recently, direct electrical stimulation of the peripheral VN in the liver of endotoxemia rats was found to inhibit TNF-a synthesis, attenuate peak serum TNF-a levels, and prevent the development of shock [25]. Similarly, vagotomized mice with experimental pancreatitis exhibited significantly worse tissue damage relative to mice with intact VNS. The severity of the pacreatitis was significantly decreased after administration of GST-21 and a7nAchR receptor agonists [26]. In this study, we found that VNS decreased the level of pro-inflammatory cytokines (TNF-a, IL-1β, and IL-6) 24 h after reperfusion in the ischemic penumbra.

The cellular and molecular mechanisms for anti-inflammation are partly attributable to acetylcholine (Ach), a neurotransmitter mainly released from VN endings. Activation of a7nAChR by Ach on macrophages suppresses the release of pro-inflammatory cytokines in peripheral circulation, thereby preventing tissue damage via the inflammation reflex of the VN. Although it had identified that VNS was effective due to recruitment of the cholinergic anti-inflammatory signaling pathway in the periphery system [27], there was little evidence available regarding its link to neuro-inflammatory modulation in the central nervous system (CNS).

The signals triggered by VNS elicit the endogenous cholinergic anti-inflammatory pathway by activating a7nAchR. These receptors are commonly expressed in brain, including neurons,glia, and endothelial cells; and activation of these receptors can enhance neuronal resistance to ischemic or other types of insults. This is the so-called “cholinergic anti-inflammatory pathway,” and it represents a physiological mechanism whereby the brain can interact with the innate immune system to control inflammatory response [28].

After an ischemic insult, resident microglia and astrocytes activate the CNS immune response, produce pro-inflammatory cytokines, and upregulate the production of reactive oxygen species (ROS) by NADPH oxidase (detrimental for stroke outcome). TNF-a and IL-1β in brain tissue are primarily produced by microglia and other immune cells [29] that rapidly respond to local injury. In recent studies, growing evidence has shown that activation of a7nAchR expressed on microglia is neuroprotective and an integral party in the fight against various brain injuries. In addition, microglia a7nAchR activation may afford neuronal protection against ischemic injury in vitro models of oxygen and glucose deprivation [30]. Wang et. al [31] found that although VNS inhibited cytokine release in wild type littermates, it had no effect on the prevention of TNF-a release in a7nAchR knockout mice. Immunofluorescence staining for a7nAchR and microglia cells partly overlapped, suggesting that VNS suppression of pro-inflammatory cytokines production was in part due to activation of a7nAchR expressed on microglia cells.

The PI3K/Akt signaling pathway plays a crucial role during cellular development, function, and survival [32]. Akt is considered to be cytoprotective through intrinsic mechanisms during cell injury [33], and p-Akt can activate downstream caspases, which is associated with the release of cytochrome, the activation of caspase 9, and activation of caspase 3. Previous reports had shown that the hepatic protective effect of nicotine was associated with a suppression in peripheral inflammation via activation of the vagal cholinergic pathway, increased expression of the anti-inflammatory protein heme oxygenase-1,and downstream activation of Akt [34]. In acute myocardial ischemia, vagal nerve stimulation increased Hypoxia-inducible factor-1 alpha (HIF-1a) expression and reduced infarct size [35]. Krafft PR et al. [36] demonstrated that that a7nAchR activation decreased neuronal cell death by increasing p-Akt and decreasing cleaved caspase 3 expression in a mouse model of intracerebral hemorrhage. Interestingly, nicotine via activation of the α7nAChR receptor in the pro-survive pathway is neuroprotective in PC12 cells [37]. In order to assess the anti-apoptotic mechanism of VNS in acute cerebral I/R models, we evaluated protein expression of a7nAchR, p-Akt, and caspase 3 at 24 h after ischemic stroke.

From our study, we observed that the protein level of both a7nAchR and p-Akt was significantly higher in the I/R+VNS group than the I/R group and that the expression of cleaved caspase-3 was significantly less in the I/R+VNS group. Thus, the protective effect provided by VNS in ischemic brain tissue may be partly due to increased expression of a7nAchR and p-Akt and decreased activation of proapoptotic caspase. Similar findings were demonstrated in a rat model of subarachnoid hemorrhage following a7nAchR stimulation [38]. Taken together, these data suggested that activation of the endogenous cholinergic pathway led to anti-inflammation and prevention of apoptosis partly by activation of a7nAchR expressed on microglial cell and a7nAchR/Akt pathway in brain, respectively. Therefore, activation of a7nAchR, either electrically with VNS or pharmacologically, could be a novel therapeutic method for early intervention in preventing secondary injury in acute ischemic stroke.

However, there are several limitations of our study. We only used an acute cerebral I/R model and did not confirm our findings at later time points in a chronic cerebral I/R model. To date, there is a little evidence available to suggest that neuroplastic processes could be enhanced by VNS in the rat brain during the recovery phase following stroke [39] and if it might be an effective therapy to restore motor function clinically. Interestingly, some studies have found that acute VNS increased the expression in cortex and hippocampus of rat of brain-derived neurotrophic factor and fibroblast growth factor in the hippocampus and cortex of rat, both beneficial for enhancing neurogenesis and functional recovery [40]. In addition, our methods for the assessment of neurological deficit score were not ideal and could be improved upon in subsequent studies.

## Conclusions

We showed that VNS could modulate the inflammatory responses in a rat model of acute cerebral I/R injury by activation of a7nAchR expressed on microglia. Furthermore, the a7nAchR/Akt pathway may be involved in the anti-apoptotic processes induced by VNS. Although there are also other mechanisms responsible for VNS-associated neuroprotection after ischemic stroke, the “endogenous cholinergic pathway” likely plays an important role in brain in response to ischemic insults.
